# The Microenvironment in Epstein–Barr Virus-Associated Malignancies

**DOI:** 10.3390/pathogens7020040

**Published:** 2018-04-13

**Authors:** Geok Wee Tan, Lydia Visser, Lu Ping Tan, Anke van den Berg, Arjan Diepstra

**Affiliations:** 1 Molecular Pathology Unit, Cancer Research Centre, Institute for Medical Research, Jalan Pahang, 50588 Kuala Lumpur, Malaysia; geokwee@imr.gov.my (G.W.T.); luping@imr.gov.my (L.P.T.); 2 Department of Pathology and Medical Biology, University of Groningen, University Medical Center Groningen, Hanzeplein 1, code EA10, P.O. Box 30.001, 9700 RB Groningen, The Netherlands; l.visser@umcg.nl (L.V.); a.van.den.berg01@umcg.nl (A.v.d.B.); 3 Department of Medical Sciences, School of Healthcare and Medical Sciences, Sunway University, Jalan Universiti, Bandar Sunway, 47500 Selangor Darul Ehsan, Malaysia

**Keywords:** Epstein-Barr virus, tumor microenvironment, Hodgkin lymphoma, undifferentiated nasopharyngeal carcinoma, gastric carcinoma, immune escape, susceptibility

## Abstract

The Epstein–Barr virus (EBV) can cause a wide variety of cancers upon infection of different cell types and induces a highly variable composition of the tumor microenvironment (TME). This TME consists of both innate and adaptive immune cells and is not merely an aspecific reaction to the tumor cells. In fact, latent EBV-infected tumor cells utilize several specific mechanisms to form and shape the TME to their own benefit. These mechanisms have been studied largely in the context of EBV+ Hodgkin lymphoma, undifferentiated nasopharyngeal carcinoma, and EBV+ gastric cancer. This review describes the composition, immune escape mechanisms, and tumor cell promoting properties of the TME in these three malignancies. Mechanisms of susceptibility which regularly involve genes related to immune system function are also discussed, as only a small proportion of EBV-infected individuals develops an EBV-associated malignancy.

## 1. Introduction

Epstein–Barr virus (EBV) is a causal factor in various malignant diseases which usually originate from B cells or epithelial cells. Much is known about the transformative properties of latent EBV infection in the tumor precursor cells in these cancers. In addition to the possession of tumor cell-transforming properties, EBV can also influence the composition and the properties of the cells present in the tumor microenvironment (TME). The TME consists of non-transformed cells that are predominantly immune cells, giving the TME a reactive, inflammatory appearance. In addition, the TME contains chemokines, cytokines, and other bio-active substances. The TME is critically important in disease pathogenesis as the tumor cells need to evade anti-EBV immune responses and, in many instances, even receive support from TME cells. As only a small fraction of latent EBV-infected cells develop into a malignancy in a minority of EBV-infected individuals, it is likely that characteristics of the host immune system are associated with susceptibility, i.e., the likelihood of EBV-infected cells to develop into a malignancy.

In some EBV-associated cancers, little data on the TME is available because of low incidence of the disease (e.g., NK/T cell lymphoma), a minor TME component (e.g., Burkitt lymphoma), or a particularly heterogeneous immune deficiency (e.g., post-transplant lymphoproliferative disorder). In this review, we will discuss the composition and the function of the TME in the three most widely studied EBV-associated cancers, i.e., classical Hodgkin lymphoma (HL), undifferentiated nasopharyngeal carcinoma (NPC), and gastric carcinoma (GC). In western populations, approximately 30% of the HL cases are EBV positive (EBV+), whereas higher percentages are observed in tropical regions [1]. Virtually all undifferentiated nasopharyngeal carcinoma cases are positive for EBV [2], while only approximately 10% of gastric carcinoma cases are EBV+ [3]. As the interactions among EBV, tumor cells, and TME are numerous and intricate, we do not aim to give a comprehensive review of all published (possible) interactions. Rather, we will present an overview of mechanisms which we believe are most relevant in terms of impact and frequency. The mechanisms EBV uses to influence the TME will be described as well as mechanisms of immune escape and TME-induced tumor cell-promoting mechanisms. In addition, we will discuss studies on genetic susceptibility.

## 2. Composition of the Microenvironment

**Figure 1:**
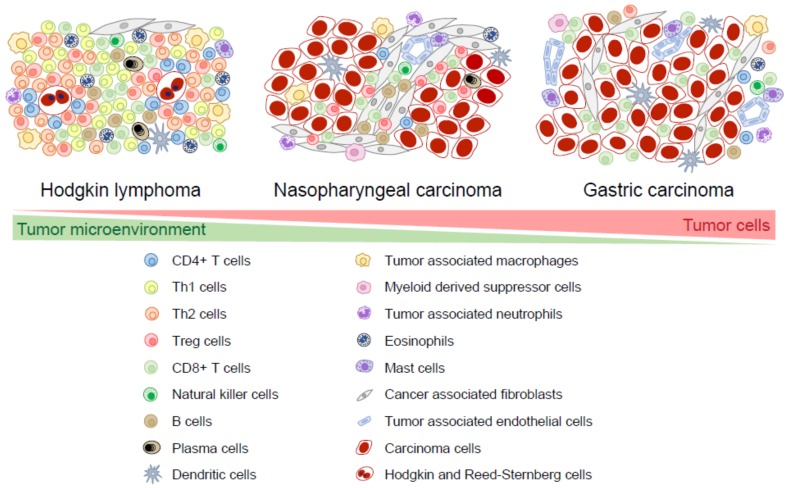
Composition of the tumor microenvironment in EBV-associated malignancies. The cellular composition of the microenvironments of typical cases of EBV+ Hodgkin lymphoma, undifferentiated nasopharyngeal carcinoma and EBV+ gastric carcinoma are shown.

There is a large overlap in tumor cell-TME interactions between EBV+ and EBV-negative cancers in both HL and GC and it is not always possible to indicate if certain characteristics are EBV specific. Therefore, we only state the differences between the TME of EBV+ and EBV-negative cases when there is available data. EBV+ HL, NPC, and EBV+ GC are often heavily infiltrated with immune cells, such as T cells, B cells, natural killer cells, dendritic cells, macrophages, myeloid-derived suppressor cells, neutrophils, eosinophils, and mast cells. Other non-immune cells derived from mesenchymal stem cells, such as fibroblasts and endothelial cells, are also present in the TME [4,5]. In HL, the TME cells usually outnumber tumor cells by >100:1 and EBV+ HL tend to have a somewhat larger TME component than EBV-negative HL [1]. In EBV+ GC, the TME component is larger than in EBV-negative GC [6]. It should be noted that both the size and the composition of the TME in EBV+ HL, NPC, and EBV+ GC can be significantly variable among individual cases. This probably reflects different levels of attraction of the TME cells by chemokines as well as different levels of activation by cytokines and cell-to-cell interactions. Here, we describe the cells and chemokines which can be present in the TME. Whenever relevant, we make a distinction between tumor cell areas and stromal areas. The latter comprises areas of connective tissue, often with some degree of fibrosis. A schematic representation of TME cells in typical cases of EBV+ HL, NPC, and EBV+ GC is shown in Figure 1.

### 2.1. T Cells

In HL, T cells form distinct layers around the tumor cells, called rosettes. Most of these rosetting T cells are CD4 positive (CD4+), consisting of either T helper 2 (Th2) cells that are involved in inducing humoral immune responses or regulatory T (Treg) cells that mediate immune suppression [7,8,9]. A number of chemokines from the CC sub-family are involved in the recruitment of Treg cells and Th2 cells in the TME and are probably responsible for the formation of the rosettes. These chemokines, namely CCL17 (TARC), CCL20 (MIP3A), and CCL22 (MDC), are expressed by Hodgkin tumor cells [10,11]; in EBV+ HL, expression of CCL20 is induced by Epstein–Barr virus nuclear antigen 1 (EBNA1) [11]. Both CCL17 and CCL20 attract CC chemokine receptor 4 positive (CCR4+) Treg and Th2 cells to HL tumor cells in vitro [12]. CD4+ Th1 cells can induce cytotoxic immune responses and CD8+ T cells are located outside the rosettes, further away from the Hodgkin tumor cells as compared to the Th2 and Treg cells [5,13,14]. The presence of EBV elicits a higher number of infiltrating CD4+ T cells [8] and CD8+ T cells [15], including activated cytotoxic CD8+ T cells (CTLs) [13,15]. This can be explained by the tumor cell-specific production of the CXC-family chemokines CXCL9 and CXCL10 which attract CXCR3+ Th1 cells [16,17]. EBV is also associated with the presence of additional T regulatory 1 (Tr1) cells [8] which are induced Treg cells that secrete IL-10. In contrast, the number of natural thymus-derived Treg cells is not associated with EBV status in HL [7,13].

In NPC, T cells are the main component of the TME [18,19,20]. Natural Treg cells and CD8+ T cells are frequently present in the stroma and occasionally in the tumor cell area [19,21]. It appears that only a subset of the CD8+ T cells has cytotoxic potential [21]. Not much is known about the precise composition of CD4+ T cell subsets (Th1, Th2, Treg cells, etc.) in NPC. Th1 cells are present and attracted by CXCL9 [22] and CXCL10 [16]. Some NPC cases contain CCL20-expressing tumor cells which attract CCR6-expressing Treg cells, as has been shown both in vitro and in vivo [23,24]. In contrast to HL, CCL17 expression is absent in NPC [25] and, to the best of our knowledge, CCL22 expression has not been explored in NPC.

In GC, T cells are distributed among tumor cells and are also present in the stroma [26,27,28,29]. Similar to EBV+ HL, EBV triggers a significantly higher infiltration of CD8+ T cells and CTLs in EBV+ GC [6,28,29,30,31]. The number of infiltrating CD8+ T cells is always more abundant in EBV+ GC than the number of infiltrating CD4+ T cells, often up to a ratio of 10:1 [6]. RNA-seq analysis of GC samples revealed a significant association of a CTL signature with EBV load [32]. GC cells secrete CCL20 [33], CCL22 [31], and CCL17 (in a minority of cases) [31]. EBV+ GC cells produce more CCL22 and attract more Treg cells than EBV-negative GC in vitro [31].

In short, T cells are an important component of the TME in the three EBV-associated malignancies and these T cells are actively recruited by chemokines that are produced by the tumor cells. The numbers of different subsets within the T cell population varies substantially between individual cases. Although there is some overlap, between EBV+ and EBV-negative cases, there is an overall tendency of higher involvement of Th1 and CD8+ cells in EBV-associated diseases; this is most prominent in EBV+ GC.

### 2.2. Natural Killer Cells

Natural killer (NK) cells can rapidly kill target cells, including virus-infected cells; however, they have not been widely studied in the TME of EBV-associated malignancies. Significantly higher percentages of NK cells were reported in seven cases of EBV+ HL as compared to seven cases of EBV-negative HL (8% vs. 1%) [13]. In NPC, only a low number of NK cells is present, ranging from 1% to 9.5% and are located mainly in the stroma [18,20,34]. In EBV+ GC, NK cells are undetected [6]. The number of NK cells in EBV-associated cancers is perhaps lower than expected which might be a result of the inhibition of their recruitment and activation.

### 2.3. B cells and Plasma Cells

Upon activation, B cells can mature into plasma cells and secrete antibodies to tag pathogens or infected cells for immune destruction. In EBV+ HL, NPC, and EBV+ GC, B cells are present but usually in lower numbers than T cells. In HL, B cells, and plasma cells can be present, but it is unclear to what extent these cells are specifically attracted or resident cells of the involved lymph node. In NPC, B cells are present at varying percentages [18,19,20,35] and they mostly reside in the stroma [19,36]. In EBV+ GC, only scattered B cells are found, usually in the stroma [6,26]. Little is known about the role of these seemingly normal B cells in the TME of EBV-associated cancers.

### 2.4. Dendritic Cells

Dendritic cells (DCs) are professional antigen presenting cells that present antigenic peptides to CD4+ T cells in the context of Human Leukocyte Antigen (HLA) class II. In cancers, the activation and maturation of DCs into different functional subsets depends largely on the cytokine milieu in the TME. In EBV+ HL and NPC, high numbers of DCs are found to infiltrate tumor cell areas [37]. In NPC, mature DC cells are seen predominantly within the tumor cell area with a few in the surrounding stroma [38,39]. Higher numbers of DCs are present in EBV+ GC compared to EBV-negative GC [34]. In EBV+ GC, mature DCs are in close proximity to tumor cells and appear to have a positive correlation with the abundance of lymphocyte infiltration [40].

### 2.5. Tumor-Associated Macrophages

Macrophages polarize into diverging phenotypes with different functions depending on signals from the microenvironment. Macrophages activated by IFN-γ and lipopolysaccharide are classified as M1 macrophages with a pro-inflammatory function that supports Th1 responses (CD8+). In contrast, M2 macrophages exhibit anti-inflammatory functions and promote Th2 responses (humoral). Tumor-associated macrophages (TAMs) are macrophages that are recruited to the TME by chemokines such as CCL2 (MCP-1) and CCL5 (RANTES) [41,42]. In HL, significantly higher numbers of TAMs are observed in EBV+ HL as compared to EBV-negative HL [43,44,45,46,47]. In NPC, M1-polarized macrophages are found in both tumor cell areas and stroma [48,49], while M2-polarized macrophages, when present in high density, are often distributed in the stroma [50]. Latent membrane protein 1 (LMP1) regulates the expression of CCL2 and CCL5 in NPC cells in vitro [51], both of which can recruit TAMs. In GC, macrophages are more commonly seen in the stroma [26] and one study reported a significantly lower number of TAMs in EBV+ GC as compared to EBV-negative GC [52].

### 2.6. Myeloid-Derived Suppressor Cells 

Myeloid-derived suppressor cells (MDSCs) are derived from immature myeloid cells which failed to differentiate into mature cells of myeloid lineage, such as macrophages, dendritic cells, and granulocytes. MDSCs exhibit strong immunosuppressive activities. Circulating MDSCs are detected in the blood of HL [53,54], NPC [55,56], and GC patients [57,58,59]. MDSCs have been observed in the stroma of NPC [55] and in GC [59,60,61]. In NPC, LMP1 is associated with the presence of MDSCs, and the function of LMP1 in inducing MDSCs was validated in vitro [62].

### 2.7. Granulocytic Cells

Granulocytic cells consist of neutrophils, eosinophils, and mast cells. Varying numbers of neutrophils are present in HL and they are attracted by IL-8 which is produced by reactive cells including TAMs. Neutrophils are also associated with tumor cell necrosis probably because of their main function as phagocytic cells. In NPC, neutrophils are commonly present in the stroma [34] and one study reported the presence of tumor-associated neutrophils in approximately 10% of NPC cases [63]. In EBV+ GC, some cases have a low number or no neutrophils while some have neutrophils present at extreme numbers [64]. Eosinophils are granulocytes that are usually involved in immune responses against parasitic infection and allergy [65]. The presence of eosinophils in the TME is fairly common in cancers. Tissue eosinophilia is reported in a subset of HL, NPC, and GC cases [43,66,67,68,69,70,71]. In HL, it is more prominent in EBV-negative cases, but it can also occur in EBV+ HL [67].

Mast cells are granulocytes present in tissue which release histamine in allergic reactions. In EBV+ HL, mast cells are scattered diffusely within the lymphocyte background [72]. Higher numbers of mast cells are more commonly seen in the NS subtype [66,72,73]. This corresponds to the association of the NS subtype with fibrosis; mast cells are involved in promoting fibrosis [74]. In NPC, mast cells are observed in the stroma [34,75]. In GC, mast cells are distributed near blood vessels and may play a role in angiogenesis [76,77].

### 2.8. Cancer-Associated Fibroblasts

Cancer-associated fibroblasts (CAFs) are activated fibroblasts in the TME that have a disparate immunophenotype compared to quiescent fibroblasts. In HL of the NS subtype, fibroblasts are involved in forming fibrous septae. In NPC, CAFs surround tumor cell nests [78] and their density varies among NPC cases [79]. CAFs are also present in EBV+ GC [80]. In epithelial malignancies, CAFs may play an important role in promoting tumor progression, e.g., by secreting proteases that degrade the extracellular matrix [81,82].

### 2.9. Endothelial Cells

Endothelial cells form a layer on the internal surface of blood and lymphatic vessels and can be involved in inflammation, fibrinolysis, and angiogenesis [83]. In tumor neoangiogenesis, they are activated by signals from tumor cells such as VEGF. In EBV+ HL, VEGF is produced by the tumor cells in the majority of cases [84]. A study on NPC demonstrated that VEGF expression is induced by LMP1 [78,85]. In addition to their main role in neoangiogenesis, endothelial cells can also promote recruitment of T cells when they are stimulated by Hodgkin tumor cell-derived lymphotoxin-α, also known as tumor necrosis factor-β (TNF-β) [86].

## 3. Immune Escape Mechanisms

Individuals who are latently infected by EBV have mounted adaptive, T cell-dependent anti-viral immune responses that keep the number of infected host cells low. In the majority of individuals, this prevents the development of EBV-driven malignancies. These malignancies can only occur if the tumor precursor cells have escaped from these T cell responses. Many escape mechanisms are present at the time of diagnosis considering the T cell-rich TME. These mechanisms are related to antigen presentation by HLA as well as antigen-dependent activation of T cells. The complex antigen-dependent interaction between tumor cells and T cells can be affected at many different levels, as explained in more detail below.

### 3.1. Latency Expression Patterns 

EBV+ HL, NPC, and EBV+ GC display a type II latency expression pattern of EBV proteins that includes expression of EBNA1 and LMP2. LMP1 is always expressed in EBV+ HL, at variable levels in NPC (50–80%) [87], and is usually absent in EBV+ GC (10%) [88]. BARF1 is highly expressed in NPC and EBV+ GC [89] but not in EBV+ HL [90]. The immunodominant EBNA3A, 3B, and 3C proteins are not expressed; this can be considered as a mechanism of immune escape. Another well-known immune escape mechanism is the glycine-alanine repeat domain that is present in the EBNA1 protein; this protein inhibits efficient ribosomal translation and strongly inhibits its peptides from being presented through HLA class I [91]. However, EBNA1-specific CTL responses do occur in EBV+ cancer patients, albeit at low frequencies [92]. In addition, EBNA1-specific CD4+ T cell responses are common and appear to be effective in killing EBV-infected cells [93]. Although T cells targeted to LMP1 occur infrequently, LMP2-specific CD8+ T cells are often detectable at the time of diagnosis in NPC [94]. In short, although the EBV+ tumor cells are not as immunogenic as lytically infected cells, they are still capable of eliciting EBV-specific immune responses. In addition, these tumor cells are also threatened by immune responses directed to neoantigens, i.e., antigens derived from proteins that have been altered, such as by gene mutations.

### 3.2. Expression of HLA

One straightforward mechanism employed by tumor cells to prevent the presentation of antigenic peptides is to downregulate expression of HLA on the tumor cell membrane. This occurs in EBV+ HL in which ~30% of cases the tumor cells have lost expression of HLA class I and ~30% of cases have lost expression of HLA II (only partly overlapping) [95]. This is fewer than is seen in EBV-negative HL; this is surprising because one would expect that anti-EBV immune responses would result in a stronger selective pressure to downregulate HLA. However, it has been proposed that HLA expression is necessary for the surrounding lymphocyte infiltration which can nurture tumor cell growth as well as survival. HLA class I and II are even more strongly expressed in Hodgkin tumor cells in ~40% of EBV+ HL cases when comparing with normal B cells. This highly increased expression may be induced by LMP1 or by changes in chromatin organization by PML (that can be disrupted by EBNA1) and/or special AT-rich sequence binding protein 1 (SATB1) [96,97]. There is less data on NPC, but it has been reported that complete loss of HLA class I expression occurs in approximately 20% of cases and partial loss in an additional 58% of cases [98]. Another study showed a loss of HLA class I expression in 50% of NPC cases [99]. Other changes in the antigen presenting machinery may alter the quality and quantity of presented peptides as well. This has been shown for HLA-DM, a crucial molecule in the presentation of antigens in the HLA class II pathway. HLA-DM is necessary for the displacement of the invariant chain CLIP peptide from HLA class II to allow binding of antigenic peptides. In the absence of HLA-DM, HLA class II will be expressed at the cell surface but only with the non-immunogenic CLIP protein. In 40% of EBV+ HL, HLA-DM expression is missing while cell surface HLA class II expression is present [95], indicating that the HLA class II expression is not functional. In HL, another mechanism that probably prevents antigen recognition by CD8+ T cells is the formation of CD4+ T cell rosettes. These rosettes form a physical barrier between CD8+ T cells and the tumor cells.

### 3.3. Co-Stimulation

In addition to antigen recognition, T cells need a second co-stimulatory signal to become fully activated. This second signal can also be inhibitory. These so-called immune checkpoints, are important in maintaining immune homeostasis. Co-inhibitory receptors, such as programmed death 1 (PD-1), cytotoxic T lymphocyte antigen 4 (CTLA-4), and lymphocyte activation gene 3 (LAG-3), control T cell responses. Many tumors take advantage of the co-inhibitory receptors to suppress anti-tumor responses. Interaction of PD-1 with its ligands PD-L1/PD-L2 inhibits T cell proliferation and induces apoptosis of tumor-specific T cells. It also promotes differentiation of CD4+ T cells into Tregs and thereby increases the resistance of tumor cells against attack from CTLs. PD-L1 has been shown to be highly expressed in tumor cells and TME cells including TAMs in EBV+ HL, NPC, and EBV+ GC [100,101,102,103]. The increased expression of PD-L1 and PD-L2 is partially caused by the amplification of chromosome 9p24.1 in EBV+ HL and EBV+ GC [104,105]. In addition, LMP1 has been shown to upregulate PD-L1 expression in NPC [103]. CTLA-4 also negatively regulates T cell activation by binding to the T cell costimulatory receptors CD80 and CD86. In NPC, CTLA-4 can be overexpressed by tumor cells [106]. Thus, overexpression of PD-L1/PD-L2 and CTLA-4 can induce T cell exhaustion and is a means of immune escape in EBV-associated cancers.

### 3.4. Excretion of Immunosuppressive Agents

IL-10 is an immunosuppressive cytokine that inhibits Th1 cells and CTLs. Expression of IL-10 has been shown to be higher in EBV+ HL, NPC, and EBV+ GC compared to their EBV-negative counterparts [107,108,109,110]; higher secretion of IL-10 corresponds to lower numbers of CTLs in NPC and EBV+ GC [109,111]. In EBV+ malignant B cells, LMP2A induces expression of IL-10 [112]. In NPC, one study showed the presence of IL-10-producing B cells in the TME [35]. Moreover, NPC cells express annexin II, a ligand for DC-specific intercellular adhesion molecule-3-grabbing nonintegrin (DC-SIGN) to stimulate increased production of IL-10 by DCs [113]. IL-10 can also be produced by Tr1 cells, TAMs, and MDSCs. Another immunosuppressive cytokine TGF-β can induce Treg and Th17 cell differentiation from naïve CD4+ T cells as well as polarize both macrophages and neutrophils to support tumor cells [114]. Blockade of TGF-β can increase the activation of CD8+ T cells into CTLs [114]. In HL, TGF-β is produced by tumor cells [115] and by MDSCs which are activated through a constant stimulation by the tumor cells [116]. Galectin-1 is a soluble beta-galactoside binding lectin that is secreted in about half of EBV+ HL cases as well as in NPC cases [117]. It reduces infiltration of CD8+ T cells in the TME and impairs immune responses against LMP1 and LMP2 [118]. In NPC, galectin-9 produced by tumor cells was shown to induce apoptosis in Th1 cells [119]. Overall, immune suppressive agents produced by tumor cells and/or by cells in the TME are important components of EBV-associated diseases.

### 3.5. Innate Immune Responses

In addition to escaping from antigen-specific immune responses, EBV+ tumor cells also need to escape from innate immune responses. EBV+ tumor cells can prevent adaptive immune recognition by downregulation of HLA class I; however, these tumor cells are then at risk of NK cell-mediated killing. Indeed, NK cells can sense the loss of HLA expression by lack of ligation of killer inhibitory receptors. HLA-G is a non-classical HLA molecule that can inhibit NK cell activation when expression of classical HLA class I is lost. HLA-G expression has been observed in HLA class I negative EBV + HL cases [120] and in NPC as well [121]. Indoleamine 2,3-dioxygenase (IDO1) expression can be regulated by the Epstein–Barr virus-encoded small RNAs (EBERs) and has an immune suppressive effect on NK cells as well as CTLs [32]. In addition, PD-L1, IL-10, and TGF-β contribute to NK cell inhibition [122].

## 4. Tumor Cell Promoting Mechanisms

There is sufficient evidence that the tumor cells in EBV+ cancers depend on the TME for certain stimulatory factors. It is extremely difficult in both EBV+ HL and NPC to make xenografts of primary tumor tissue, indicating that the tumor cells need contacts and factors from their microenvironment. It is likely that tumor cell promoting factors are already important during the early stages of pathogenesis. Subsequently, mutations can occur that ensure constitutive activation of signalling pathways, e.g., involving nuclear factor kappa b (NF-κB) and TNFAIP3 in EBV+ HL [123,124,125,126]. Mutations in the NF-κB pathway also occur in NPC [127,128] and lead to constitutive activation of NF-κB. The most important pathways involved in tumor cell promotion in EBV-associated malignancies are the NF-κB, the janus kinase/signal transducers and activators of transcription (JAK/STAT), and microtubule-associated protein kinase/extracellular signal-regulated kinase (MAPK/ERK) pathways. Significant redundancy exists in the mechanisms that activate these pathways, emphasizing their important role in pathogenesis.

### 4.1. Stimulation of NF-κB

The NF-κB pathway can be divided into canonical and non-canonical pathways. The canonical pathway promotes inflammation, cell proliferation, and cell survival; it also involves the B cell receptor (BCR), Toll-like receptors, and TNF family receptors. RelA is a specific component of this pathway. Normally, activation of B cells is triggered by the binding of the BCR to antigen. Loss of BCR expression is lethal to normal B cells but occurs in HL tumor cells. The EBV-encoded LMP2A is a functional homologue of the BCR and may thus rescue the tumor cells in EBV+ HL. LMP2A is also known to be expressed in NPC [129,130] and in a proportion of EBV+ GC as well [131]. Decoy receptor 3 (DcR3) is a member of the tumor necrosis factor superfamily (TNFSF) family which can bind to TNFSF14 and FasL as well as prevent apoptosis. DcR3 is expressed in HL but no association with EBV was found [132]. In contrast, 75% of LMP1+ NPC tissues overexpress DcR3. DcR3 is upregulated in vitro by LMP1 and enhances migration and invasion via PI3K and NF-κB [133]. The non-canonical NF-κB pathway regulates lymphoid development, has anti-inflammatory activity, is activated via CD30 and CD40 amongst others, and is dependent on RelB. Overexpression of CD30, which is ubiquitous in EBV+ HL tumor cells [134], can lead to spontaneous NF-κB activation [135]. In addition, CD30L is found on eosinophils and mast cells in HL and may activate CD30 by cell-to-cell interactions [136,137]. CD40 is expressed on the tumor cells in EBV+ HL [138,139], NPC [36], and EBV+ GC [140]. In EBV+ HL, expression of IL-10 by the tumor cells can enhance the expression of CD40L on T cells [141]. In EBV+ GC, the virus activates CD40 signalling and promotes survival and proliferation [142]. Thus, signals derived from the TME can stimulate the non-canonical NF-kB pathway. In addition, LMP1 mimics the function of CD40 and interacts with TRAFs to activate NF-κB [51,62,143].

### 4.2. Stimulation of JAK/STAT

The JAK/STAT pathway is stimulated via cytokine receptors. Upon binding of the cytokine to the receptor, dimerization takes place and JAK proteins are phosphorylated. This leads to phosphorylation and dimerization of STATs which move to the nucleus and function as transcription factors. The JAK/STAT pathway is involved in proliferation, survival, invasion, and inflammation. In EBV+ HL, the JAK/STAT pathway is constitutively activated [144] by amplification of JAK2 [145] or, in some cases, mutations in the inhibitors PTPN1 [146] and SOCS1 [147]. In addition to stimulation via NF-κB, LMP1 also interacts with JAK3 and thereby activates STAT1 in B cells [148]. Interestingly, in NPC, LMP2A downregulates NF-kB and STAT activity and represses LMP1 expression, showing that mechanisms in epithelial cells are different from those in B cells [149].

### 4.3. Cytokine Receptors

In EBV+ HL, receptors for IL-3, IL-6, IL-7, IL-9, IL-13, IL-15, and IL-21 are present on the tumor cells. Most cytokines are also produced by the Hodgkin tumor cells, whereas some are produced exclusively by other cells in the microenvironment such as IL-3 by eosinophils, mast cells, and T cells. IL-6, IL-9, and IL-21 are produced by Hodgkin tumor cells and by T cells in the TME [150,151]. In EBV+ HL, IL-13 activates STAT6 and induces expression of LMP1 [152]. EBNA1 binds to the *IL6R* at the transcription start site and induces IL-6R expression in EBV+ B cells [153]. IL-6R expression has been reported in NPC and IL-6 is produced by tumor cells in the TME, acting as a growth factor and resulting in STAT3 activation [154]. Both leukemia inhibitory factor (LIF) and its receptor (LIFR) are expressed in NPC. LMP1 enhances LIF expression thereby promoting proliferation of the tumor cells in NPC [155]. CXCR4 is expressed on the membrane of NPC cells but is also found in the nucleus, possibly playing a role in cancer development and progression [156] as well as metastasis [157]. CXCR4 expression and its translocation to the nucleus is regulated by LMP1 [158]. LMP1 induces tyrosine sulfation of CXCR4; this is likely associated with cell motility and invasiveness [157]. The ligand for CXCR4, SDF1, is also expressed in NPC [158]. Another cytokine, IL-1β, is expressed in EBV+ GC; in vitro experiments have shown that it acts as an autocrine growth factor [159].

### 4.4. Stimulation of MAPK/ERK

The MAPK/ERK pathway can be stimulated by binding of soluble or membrane bound factors to receptor tyrosine kinases (RTKs). The cascade includes different members of the MAPK/ERK family and affects proliferation and differentiation of cells. In EBV+ HL, no activating mutations in any of the RTKs have been found thus far, whereas in NPC, mutations in MAPK/ERK activators occur in 13–15% of cases [128,160]. In HL, several RTKs are usually co-expressed although less frequently in EBV+ HL [161]. Platelet-derived growth factor receptor alpha (PDGFRA) is expressed in 75% of all HL cases, including EBV+ HL, and it is activated by autocrine stimulation [162]. Discoidin domain receptor 1 (DDR1) is found in 75% of EBV+ HL cases and is induced by LMP1 [163]. Expression of the other RTKs, i.e., DDR2, EphrinB1, RON, TRKA, and TRKB, are each found in approximately 30% of cases. Type I collagen I is present in sclerotic bands in NS type HL and can bind to DDR1 as well as to DDR2 [161]. It increases survival and induces protection from apoptosis [163]. Nerve growth factor (NGF) produced by mast cells can bind to TRKA [161]. C-Met expression is found in HL [164,165], NPC [166], and EBV+ GC [167], while its ligand hepatocyte growth factor (HGF) is produced in dendritic cells in HL and in the interstitial tissue surrounding the tumor in NPC [166]. HGF is expressed in some GC, but it is unknown whether this includes EBV+ GC. Both latent EBV infection and signaling induced by CD40L on T cells can induce c-Met expression in B cell lymphoma [168,169,170]. Hodgkin tumor cells express IGF-1R [171], and IGF-1 expression can be found in NPC [172] and EBV+ GC [173]. TNF receptor signals are conveyed by TRAF1 which is expressed in 40% of NPC cases and in all LMP1+ NPC cases. EBERs can induce expression of IGF-1 in NPC and EBV+ GC cell lines and act as an autocrine growth factor [172,173]. Additional factors that are regulated by EBERs can have tumor growth supportive effects [172,173].

## 5. Susceptibility to EBV-Associated Malignancies 

The host immune response against EBV is thought to be associated with the risk of development of EBV-associated malignancies. One underlying hypothesis is that a less efficient host immune response leads to a higher number of EBV-infected cells, thus increasing the pool of potential tumor precursor cells. An alternative hypothesis is poor clearance of EBV-infected cells by the immune system during all or some stages of malignant transformation. Both potential mechanisms are present in the context of primary immune deficiency and immune suppression, e.g., in post-transplant lymphoproliferative disease. In the general population, the efficiency of the host immune response against EBV is determined in part by genetic factors [174]. This efficiency has been assessed by measuring EBV-specific antibody titers or by EBV copy numbers. Several single nucleotide polymorphisms (SNPs) have been associated to these measurements based on candidate gene approaches, e.g., variants within IL-10 [175]. Most of these associations have not been confirmed in independent studies, although associations with the HLA class II region are reported recurrently. Genome-wide association studies (GWAS) in large cohorts revealed associations of anti-EBNA-1 antibody levels with HLA-DRA, HLA-DRB9, and HLA-DRB1 amongst others [176]. In a Mexican-American family-based study, two independent loci within the HLA class II region were identified to be associated with anti-EBNA-1 antibody levels. The most likely candidates for these associations are HLA-DRB1 and HLA-DQB1 [176]. In a French family cohort of 424 individuals, high anti-EBNA-1 levels were associated with a SNP in the HLA class II region [177]. In two large studies, suggestive associations with SNPs and specific genomic regions were observed for EBV copy numbers in cohorts of more than 900 and 1700 lymphoblastoid cell lines (LCLs). These LCLs were derived from individuals with different ethnicities, and clear differences were observed for EBV copy numbers and ethnicity [178].

### 5.1. Genetic Associations in EBV+ HL

There are many studies on genetic associations with susceptibility to EBV-associated cancers and some of the observed associations overlap. For a more detailed overview of all currently available association studies, we refer to the extensive review published by Houldcroft and Kellam [174]. In 2005, we studied associations within the HLA region in EBV-stratified Dutch HL patients and showed two distinct associations, i.e., one with EBV+ HL in the HLA class I region and one with EBV-negative HL in the HLA class III region [179]. The association of EBV+ HL with the HLA class I region was confirmed in subsequent GWAS studies [180,181]. This association was shown to be related to the HLA-A gene, with HLA-A*01 as a risk and HLA-A*02 as a protective type for the development of EBV+ HL [182,183]. A modeling of HLA associations by means of direct HLA typing in combination with selected SNPs from GWAS revealed HLA-A*01 and HLA-B*37:01 as risk alleles and HLA-DRB1*15:01 and DPB1*15:01 as protective alleles [184]. As HLA-A*01-restricted immune responses to EBV latent peptides have never been encountered in the general population, the risk effect of this allele may be explained by a decreased ability of CD8+ T cells to recognize EBV-infected cells. In contrast, HLA-A*02 responses to EBV latent peptides, mainly LMP2A, are common and the protective effect of HLA-A*02 has been studied in EBV+ HL tissue. In (relatively rare) HLA-A*02 positive cases, the number of CD8+ T cells was significantly higher than in cases without HLA-A*02 [185]. In a Northern Chinese EBV+ HL population, no associations were observed for HLA-A*01 and HLA-A*02 using 2-digit HLA typing. Additional sequence-based typing indicated that the HLA-A*02:07 allele predisposes to EBV+ HL, while the other HLA-A*02 alleles do not [186].

### 5.2. Genetic Associations in NPC

As with EBV+ HL, the most striking associations in GWAS studies in NPC are found within the HLA region, with HLA-A as the most prominently predicted candidate gene [187,188,189,190]. Several studies confirmed the strong association with HLA-A, e.g., a study in Chinese NPC cases showing a strong association with HLA-A and HCG9 [190]. Focusing specifically on the HLA region, the associations were linked to the peptide binding motif of HLA-A*11:01, with a second independent association driven by HLA-B*13:01, B*38:01, and B*55:02 [191]. The HLA-A*02:07 allele, which is common in Eastern Asia, has also been shown to predispose to the development of NPC [192]. In a more recent study, the associated HLA-A amino acid variants and SNPs confirmed the correlation with HLA-A*11:01 and, to a lesser extent, with HLA-A*02:07 [188]. Interestingly, the HLA-A*02:07 association with NPC is shared with EBV+ HL in Chinese populations, but there is no association with HLA-A*01, probably a result of the low frequency of this HLA-A type in Chinese populations. A second gene mapping close to the HLA-A gene, the G-protein coupled receptor subunit gamma aminobutyric acid b receptor 1 (GABBR1), has also been linked to NPC susceptibility [193]. However, because of a strong linkage in the HLA region, it is not clear whether this is an independent association or not. Other non-HLA loci identified in smaller NPC cohorts include integrin-α 9 (ITGA9) and the IL1 receptor antagonist (IL-1RN). Variants in ITGA9 are strongly associated with a risk of NPC in a Malaysian Chinese population [194]. For some integrins, a link with EBV cell entry and infection have been proposed [195] which might explain the observed association with ITGA9. In a Portuguese population of NPC cases, a strong association was observed with variants in IL-1RN, which is an endogenous inhibitor of the inflammatory interleukin 1β [196]. In a search for additional non-HLA loci, the top 1000 SNPs identified by Bei et al. [187] were validated in two steps, using more than 2000 cases in the first step and more than 7000 in the second step [197]. This revealed two associated loci: TERT-CLPTM1L, which is involved in telomere function, and CIITA, the master regulator of HLA class II expression. A meta-analysis on four NPC GWAS studies also found the TERT-CLPTM1L association [198]. Another recent study reported pathogenic heterozygous germline variants in the macrophage-stimulating 1 receptor (MST1R) gene in early onset (age ≤20 years) NPC [199]. MST1R is expressed in macrophages and ciliated epithelial cells in the nasopharynx; defects in this gene may impact innate immunity and motility of cilia.

### 5.3. Genetic Associations in Gastric Carcinoma

In gastric cancer, GWAS studies are usually performed on mixed patient groups including both EBV+ and EBV-negative cases. As only a minority of the cases are EBV+, it is difficult to dissect associations that are linked to EBV. Serological typing of HLA in 110 EBV+ and 155 EBV-negative cases revealed associations with HLA-B59, DQ3, DR9, and DR11 [200]. In a targeted gene approach study, a comparison of 30 EBV+ cases to 120 EBV-negative cases revealed an association for promoter polymorphisms at the TNF-α and IL-10 gene loci [201]. Additional targeted approach studies revealed associations with Filaggrin (FLG), a filament-associated protein that binds to keratin fibers, in EBV+ GC, and EBV-negative GC [202]. Glypican-4 (GPC4), a member of the heparan sulphate proteoglycan family, was associated with EBV+ GC [203].

Thus, with some exceptions, most of the susceptibility associations in HL, NPC, and GC involve genes that are components of the immune system, which theoretically may result in (partial) immune escape. Protective gene variants are present in a lower number of patients but can still occur, as EBV uses multiple mechanisms to escape from immune responses.

## 6. Concluding Remarks

The TME is an essential component in the pathogenesis of EBV-associated malignancies. These cancers result from a combination of failing immune control and tumor cell nurturing immune responses. Interactions between cells in the TME and tumor cells are complex and vary between individual cases with the same cancer type. On the other hand, there is also a large overlap in mechanisms among different EBV-associated cancer types, as outlined in this review. Determining which EBV-induced mechanisms are most important is challenging as none of the mechanisms are present or dominant in all cases. In addition, malignant transformation itself also will have an impact on interactions between the tumor cells and cells in the TME. Research in this field is hampered by the lack of clinically or biologically recognized precursor lesions for EBV-associated malignancies as well as the absence of both animal and in vitro models that accurately recapitulate the human in vivo situation. The interactions between TME and tumor (precursor) cells are part of a highly dynamic process in which both components adapt to each other. Thus, mechanisms that are important early in pathogenesis may have been overtaken by other mechanisms at the time of diagnosis. Nonetheless, it is important to realize that there is a high variability between cases at diagnosis, especially when designing novel treatment strategies that aim to activate or disrupt the TME as a means of eradicating EBV+ tumor cells.
